# Clinical features of pulmonary infarction in older patients with pulmonary embolism: a retrospective comparative study

**DOI:** 10.3389/fmed.2025.1689618

**Published:** 2025-11-21

**Authors:** Zhen-Chuan Xing, Yuan Yuan, Hong-Xia Zhang, Jin-Xiang Wang, Shuai Zhang

**Affiliations:** Department of Pulmonary and Critical Care Medicine, Beijing Luhe Hospital, Capital Medical University, Beijing, China

**Keywords:** pulmonary infarction, pulmonary thromboembolism, embolus, older, clinical characteristics

## Abstract

**Objective:**

To analyze the clinical characteristics of pulmonary infarction in older patients with pulmonary embolism.

**Methods:**

We conducted a single-center retrospective study of 71 patients with pulmonary infarction (PI) secondary to pulmonary embolism (PE) confirmed by CTPA between January 2016 and December 2021. Participants were stratified into older (≥65 years) and non-older (<65 years) groups. We compared baseline characteristics, laboratory/imaging findings, Wells scores, PE severity, diagnosis and treatment using chi-square and Mann-Whitney U tests.

**Results:**

PI occurred in 17.1% (71/414) of PE cases, comprising 36 older patients and 35 non-older patients. Significant differences were observed in pleuritic chest pain, hemoptysis, cardiovascular disease, cerebrovascular disease, pulmonary hypertension, and ECG changes between the two groups (*P* < 0.05). PI occurred predominantly in the right lung and in lower lobes. No significant differences were observed in infarction location, number, or imaging signs between groups. The comparison of PE risk factors and Wells scores showed no significant differences. The older group showed a higher proportion of intermediate-high risk and a lower proportion of low-risk in PE severity classification (*P* < 0.05). During the medical consultation process, older exhibited higher non-emergency/respiratory first-visit rates than younger (*P* < 0.05). Similarly high rates of antibiotic therapy were observed in both groups (*P* > 0.05).

**Conclusion:**

Older PI patients have less typical chest pain, hemoptysis or ECG changes, a high proportion of comorbid cardiovascular/cerebrovascular diseases, and a higher rate of mismatch in the first visiting department. Clinicians should recognize the atypical presentations of PI in older patients and pay increased attention to this population.

## 1 Introduction

Pulmonary embolism (PE) is one of the clinically presenting of venous thromboembolism (VTE) and is the most common acute cardiovascular syndrome in the world behind myocardial infarction and stroke (1). The annual incidence of VTE ranges from 0.75 to 2.69 per 1,000 population (2). In six major European countries, 34% of VTE patients presented with sudden fatal PTE (3). Pulmonary infarction (PI) is ischemic necrosis of lung tissue caused by PE. It is a serious complication of PE with an incidence of approximately 10–50% (4–7). While current evidence has not conclusively established a direct correlation between PI and clinical outcomes in PE patients, the radiological features of PI (exudative and parenchymal consolidation) are similar to those of other lung lesions (such as infectious pneumonia or neoplastic lesions), which may increase the difficulty of diagnosing PE and indirectly affect clinical decision-making and patient management (8, 9).

Currently, the clinical characteristics and prognosis of PI have not been fully studied, and most studies on PI were focused on the general population, with few studies on PI stratified by age. Older patients have more underlying diseases and unique pathophysiological basis (such as decreased vascular elasticity, multiple comorbidities, and poor compensatory ability), which may lead to the clinical symptoms and imaging features of PI being different from those of young patients. This study retrospectively analyzed and compared the clinical characteristics of older and non-older patients with PI, aiming to provide a scientific basis for the early identification and diagnosis of PI in the older.

## 2 Methods

### 2.1 Study population and objectives

**Figure 1:**
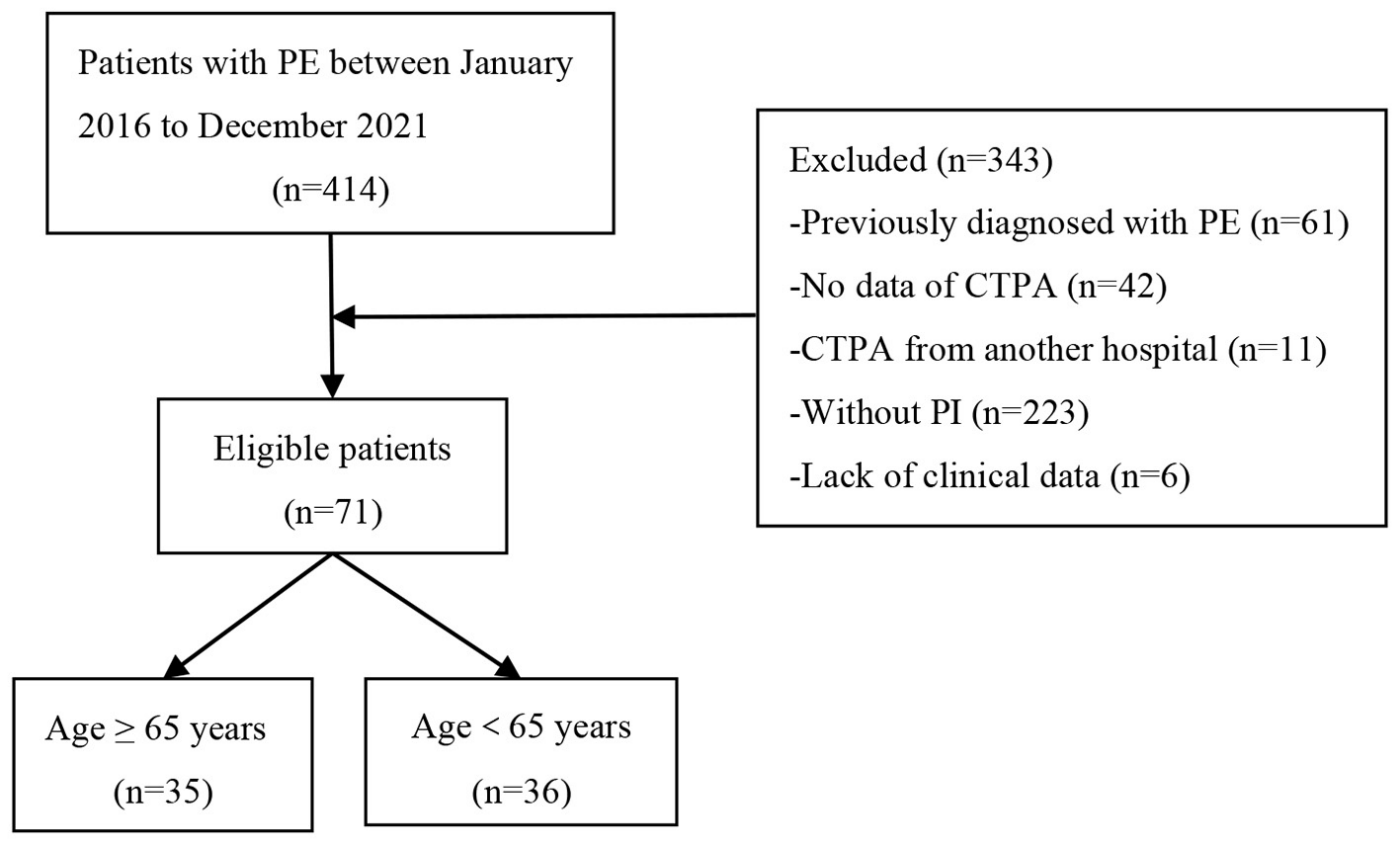
Patients flowchart.

This was a retrospective, single-center observational study conducted from January 2016 to December 2021. Patients with PE were obtained by reviewing the electronic medical record system in the Department of Pulmonary and Critical Care Medicine, Beijing Luhe Hospital, Capital Medical University, Beijing, China. The study was approved by the ethics committee of Beijing Luhe Hospital (2021-LHKY-119-02). Inclusion criteria: (i) The diagnosis of PE meets the 2019 ESC Guidelines (1); (ii) Age > 18 years old. Exclusion criteria: (i) Previously diagnosed with PE; (ii) No data of Computed Tomography Pulmonary Angiography (CTPA); (iii) CTPA from external institutions; (iv) Without pulmonary infarction (PI); (v) Lack of complete clinical data. Finally, 71 patients with PI secondary to PE were enrolled in the study. The cohort was divided into older (≥65 years) and non-older (<65 years) groups (shown in Figure 1). The aim of this study was to analyze and compare the clinical characteristics, imaging findings, risk stratification, and initial management of pulmonary infarction (PI) between older (≥65 years) and non-elgerly (<65 years) patients.

### 2.2 CT scanning

All computed tomographic (CT) scans were obtained by using a Philips 64-slice spiral CT (Toshiba) or a 256-slice spiral iCT (Philips). The patient was placed in the supine position and scanned from apex of the lung to costodiaphragmatic angle. Scanning parameters: (i) 64-slice spiral CT: tube voltage 120 kV, tube current 118 mA, pitch 1.48, gantry rotation speed 0. 5s/r, slice thickness 1 mm, slice interval 1 mm, collimation width 64 × 0.5 mm, reconstruction thickness 3 mm, slice interval 1 mm; (ii) 256-slice spiral iCT: tube voltage 120 kV, tube current 140 mA, pitch 0.993, gantry rotation speed 0.5 s/r, slice thickness 0.9 mm, slice interval 0.8 mm, collimation width 128 × 0.625 mm, reconstruction thickness 5 mm, slice interval 5 mm. Using a power injector, the following was sequentially administered via antecubital vein at 5 mL/s: (i) Pre-flush with 21 mL salin; (ii) Ioversol (350 mgI/mL, total 50 mL), (iii) Post-flush with 21 mL saline. The scan was automatically triggered.

### 2.3 Definition of pulmonary infarction and image assessment

#### 2.3.1 Pulmonary infarction (PI) definition

Pulmonary infarction results from occlusion of the distal pulmonary arteries leading to ischemia, hemorrhage and ultimately necrosis of the lung parenchyma (10). It is most often caused by pulmonary embolism (PE). Pulmonary infarction is fundamentally a pathological diagnosis, with histological examination remaining the gold standard, though this is not routinely available in clinical practice.

#### 2.3.2 Image assessment

**Figure 2:**
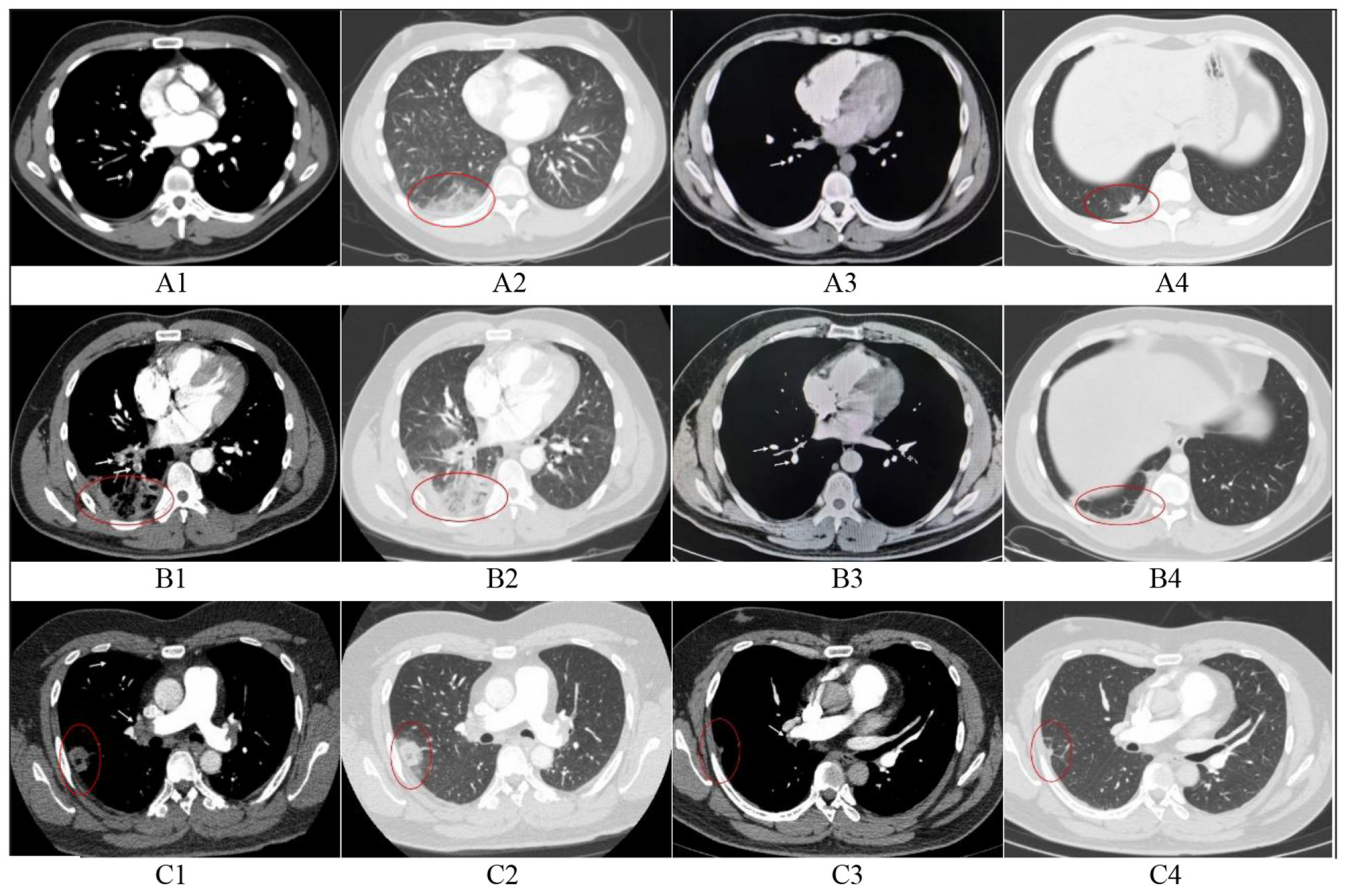
Initial and follow-up CTPA in three patients with pulmonary infarction. **(A)** A 28-year-old male. A1, Filling defect in the right lower pulmonary artery (arrow); A2, Hampton's hump in the right lower lobe, suggesting pulmonary infarction; A3, Thrombus completely resolved at 3-month follow-up; A4, Exudative opacities largely resolved, with residual scar in the right lower lobe. **(B)** A 37-year-old male. B1: Filling defect in the right lower pulmonary artery (arrow); B2, Parenchymal exudation, reversed halo sign, and ipsilateral pleural effusion in the right lower lobe, suggesting pulmonary infarction; B3, Thrombus completely resolved at 3-month follow-up; B4, Exudates absorbed, with residual fibrous strands and pleural thickening in the right lower lobe. **(C)** A 37-year-old male. C1, Filling defect in the distal right pulmonary artery(arrow); C2, Parenchymal exudation and reversed halo sign in the right upper lobe, suggesting pulmonary infarction; C3, Thrombus completely resolved at 4-month follow-up; C4, Marked absorption of exudates, with residual scar and fibrous strands in the right upper lobe. 1, Initial CTPA (Mediastinal window); 2, Intitial CTPA (Lung window); 3, Follow-up CT images (Mediastinal window); 4, Follow-up CT images (lung window).

There was no established imaging standard for a definitive diagnosis. In our study, pulmonary infarction was defined as the presence of peripheral parenchymal opacities abutting the pleura on CTPA that were distal to the occluded pulmonary artery and anatomically corresponded to the embolic focus. To enhance diagnostic certainty for pulmonary infarction, we collected post-treatment follow-up scans (CTPA or non-contrast chest CT). The resolution or marked improvement of parenchymal opacities over time confirms its association with pulmonary embolism events, further corroborating the diagnosis of PI and aiding in the exclusion of other chronic etiologies. CTPA images were independently reviewed by an experienced thoracic radiologists and senior respiratory physicians. In cases of disagreement, a final determination was reached through consensus (shown in Figure 2).

### 2.4 Clinical datas

We collected data on baseline characteristics, laboratory results, imaging findings, PE risk factors, patient-related delay, diagnostic delays, initial admitting department, Wells score, PE severity classification, treatment (antibiotics administration, thrombolysis), and mortality.

### 2.5 Statistical analysis

All data in the study were statistically analyzed by SPSS 26.0. Continuous variables with normal distribution were represented by mean ± standard deviation (mean ± SD), and inter-group comparisons were performed using independent sample *t*-test. Continuous variables with non-normal distribution were expressed as median (interquartile range) [median (IQR)], and inter-group comparisons were performed using Mann-Whitney *U*-test. Categorical variables were expressed as frequency and percentage [*N* (%)], and inter-group comparisons were performed using Pearson chi-square test (expected frequency ≥ 5) or Fisher's exact test (expected frequency <5). The significance level was set at two-sided α = 0.05.

## 3 Results

### 3.1 Baseline characteristics

Of the 414 PE cases, 141 (34.1%) were young patients (<65 years), including 35 (24.8%) with PI, while 273 (65.9%) were older patients (≥65 years), among whom 36 (13.2%) developed PI. Among the 71 patients with PI, 40 (56.3%) were male and 31 (43.7%) were female. The age of patients ranged from 19 years old to 86 years old, with a median age of (61.1 ± 17.0) years. The average hospital length of stay of the 71 patients was 11.0 (8.0, 15.0) days, and no patient died. The patient's clinical symptoms were mainly dyspnea (77.5%), pleuritic chest pain (46.5%), oedema or pain in lower limb (40.8%), cough (36.6%), and haemoptysis (29.6%). Cardiovascular (59.2%) and cerebrovascular (19.7%) disease were the most common underlying diseases (shown in Table 1).

**Table 1:** Baseline characteristics of patients with pulmonary infarction (*n* = 71).

**Patient characteristic**	**Value**
Male	40 (56.3)
Age [years, (*x* ± s)]	61.1 ± 17.0
Dyspnoea	55 (77.5)
Pleuritic chest pain	33 (46.5)
Haemoptysis	21 (29.6)
Cough	26 (36.6)
Fever	17 (23.9)
Palpitations	9 (12.7)
Syncope	8 (11.3)
Hypotension	2 (2.8)
Oedema or pain in lower limb	29 (40.8)
Cardiovascular disease	42 (59.2)
Cerebrovascular disease	14 (19.7)
Diabetes mellitus	7 (9.9)
Chronic lung disease	5 (7.0)
Malignant cancer	5 (7.0)
Liver cirrhosis	1 (1.4)
Autoimmune diseases	1 (1.4)
Nephrotic syndrome	1 (1.4)

Continuous data are presented as mean ± SD; categorical data are presented as *n* (%). Chronic lung diseases encompass chronic obstructive pulmonary disease, chronic bronchitis, bronchiectasis, pulmonary fibrosis; cardiovascular diseases encompass hypertension, coronary artery disease, arrhythmias, and heart failure.

### 3.2 Analysis of older and non-older PI patients

Among the 71 patients with PI, 36 cases were older patients (age ≥ 65 years) with a median age of 75.5 (68.3, 78.0) years, 35 cases were non-older patients (age <65 years) with a median age of 52.0 (36.0, 61.0) years. There were significant differences between the older and non-older groups in the rates of pleuritic chest pain (36.0 vs. 62.9%, *P* = 0.006), hemoptysis (13.9 vs. 45.7%, *P* = 0.003), cardiovascular disease (80.6 vs. 37.1%, *P* < 0.001), and cerebrovascular disease (30.6 vs. 8.6%, *P* = 0.020). Both groups had no statistically significant difference in gender, dyspnea, cough, fever, palpitation, syncope, hypotension, lower-leg edema or pain, and other underlying diseases (shown in Table 2).

**Table 2:** Analysis of clinical characteristics between older patients and non-older patients.

**Variables**	**Age ≥65 years (*n* = 36)**	**Age <65 years (*n* = 35)**	***χ^2^***	***P***
**Gender**				
Male	18 (50.0)	22 (62.9)	1.193	0.275
Female	18 (50.0)	13 (37.1)		
**Clinical symptom**				
Dyspnoea	28 (77.8)	27 (77.1)	0.004	0.949
Pleuritic chest pain	11 (30.6)	22 (62.9)	7.444	0.006
Hemoptysis	5 (13.9)	16 (45.7)	8.630	0.003
Cough	13 (36.1)	13 (37.1)	0.008	0.928
Fever	8 (22.2)	9 (25.7)	0.119	0.730
Palpitation	3 (8.3)	6 (17.1)	1.244	0.265
Syncope	5 (13.9)	3 (8.6)	0.502	0.479
Hypotension	0 (0.0)	2 (5.7)	2.117	0.146
Oedema or pain in lower limb	18 (50.0)	11 (31.4)	2.533	0.111
**Underlying diseases**				
Cardiovascular disease	29 (80.6)	13 (37.1)	13.843	<0.001
Cerebrovascular disease	11 (30.6)	3 (8.6)	5.418	0.020
Diabetes mellitus	2 (5.6)	5 (14.3)	1.522	0.217
Chronic lung disease	4 (11.1)	1 (2.9)	1.847	0.174
Malignant cancer	4 (11.1)	1 (2.9)	1.847	0.174
Liver cirrhosis	1 (2.8)	0	0.986	0.321
Autoimmune diseases	0	1 (2.9)	1.043	0.307
Nephrotic syndrome	0	1 (2.9)	1.043	0.307

Data are presented as *n* (%).

Comparative analysis of laboratory tests, electrocardiogram (ECG), and ultrasonographic data revealed several significant differences between the two groups. Older patients (≥65 years) demonstrated a significantly higher prevalence of pulmonary hypertension (52.8 vs. 28.6%, *P* = 0.038). The prevalence of ECG changes was significantly higher in the non-older group than in the older group (91.4 vs. 55.6%, *P* = 0.003). Specifically, the frequency of the classic S1Q3T3 pattern (22.9 vs. 0%, *P* = 0.005) and sinus tachycardia (65.7 vs. 36.1%, *P* = 0.024) was significantly higher in the younger group than in the older group. Conversely, the prevalence of atrial fibrillation was significantly higher in the older group than in the younger group (13.9 vs. 0%, *P* = 0.025). Although not statistically significant, the older group exhibited a higher proportion of elevated brain natriuretic peptide (BNP) and high-sensitivity troponin I (hs-cTnI) levels (BNP: 38.9 vs. 20.0%, *P* = 0.081; hs-cTnI: 30.6 vs. 17.1%, *P* = 0.185). The remaining parameters (including D-dimer, white blood cell count, C-reactive protein, incidence of right ventricular dilatation, and deep vein thrombosis) showed no statistically significant differences or discernible trends between the two groups (all *P* > 0.05) (shown in Table 3).

**Table 3:** Analysis of laboratory tests, eletrocardiogram and ultrasound results.

**Variables**	**Age≥65 years (*n* = 36)**	**Age <65 years (*n* = 35)**	***χ^2^***	***P***
D-Dmier (>0.243 μg/mL)	33 (91.7)	32 (91.4)	0.001	0.971
hs-cTnI (>0.015 μg/L)	11 (30.6)	6 (17.1)	1.753	0.185
BNP (> 100 pg/mL)	14 (38.9)	7 (20.0)	3.040	0.081
White blood cell (>10 × 10^9^/L)	13 (36.1)	14 (40.0)	0.114	0.736
C-reactive protein (>10 mg/L)	24 (66.7)	26 (74.3)	0.495	0.482
Pulmonary hypertension	19 (52.8)	10 (28.6)	4.304	0.038
Right ventricular dilation	23 (63.9)	21 (60.0)	0.114	0.736
Electrocardiogram changes	20 (55.6)	32 (91.4)	9.125	0.003
Sinus Tachycardia	13 (36.1)	23 (65.7)	5.079	0.024
Atrial Fibrillation	5 (13.9)	0	Fisher	0.025
Right bundle branch block	1 (2.8)	1 (2.9)	Fisher	1
T-wave inversions in V1–V6	1 (2.8)	0	Fisher	0.493
S1Q3T3	0	8 (22.9)	Fisher	0.005
Deep venous thrombosis	24 (66.7)	23 (65.7)	0.007	0.932

Data are presented as *n* (%). hs-cTnI, High-Sensitivity Cardiac Troponin I; BNP, brain-type natriuretic peptide; S1Q3T3, S wave in lead I, Q wave in lead III, and T wave inversion in lead III.

A total of 117 pulmonary infarction lesions were identified in 71 patients, including 57 lesions in older patients and 60 lesions in non-older patients. Among them, 46 patients (64.8%) had a single infarction lesion, 13 patients (18.3%) had two lesions, four patients (5.6%) had three lesions, seven patients (9.9%) had four lesions, and only one patient (1.4%) had five lesions. The distribution of infarction lesions as follows: left lung 38 cases: single upper lobe 9 cases, single lower lobe 20 cases, both lobes 9 cases; right lung 51 cases: single upper lobe 7 cases, single middle lobe 3 cases, single lower lobe 16 cases, both upper and middle lobes 1 cases, both upper and lower lobes 4 cases, both middle and lower lobes 5 cases, and all three lobes 3 cases. No statistically significant differences were found in infarction location, lesion count, or imaging signs between different age groups. The radiographic features of pulmonary infarct lesions were systematically analyzed and compared between the two groups. The most prevalent finding was the Hampton's hump, which was observed at comparable frequencies of 75.0% (*n* = 27) and 77.1% (*n* = 27) in each group, respectively, with no statistically significant difference (*P* = 0.832). Among older patients, 47.2% (*n* = 17) of subjects exhibited reversed halo sign, whereas this proportion was 28.6% (*n* = 10) in non-older patients. The difference between the two groups was not statistically significant (*P* = 0.106). Pleural effusion ipsilateral to the infarction was a common associated feature, noted in 61.1% (*n* = 22) in older patients and 40.0% (*n* = 14) of cases in non-older patients, showing a trend toward a higher occurrence in the first group that was not statistically significant (*P* = 0.075). Other features, including atelectasis (13.9 vs. 22.9%, *P* = 0.329), a semicircular shape (8.3 vs. 5.7%, *P* = 0.666), and cavity formation (2.8 vs. 2.9%, *P* = 0.984), were observed infrequently and demonstrated no significant differences between the groups (shown in Table 4).

**Table 4:** Analysis of radiographic characteristics between older patients and non-older patients.

**Variables**	**Age ≥65 years (*n* = 36)**	**Age <65 years (*n* = 35)**	***χ^2^***	***P***
**Infarct location**				
Left lung with infarction	19 (52.8)	19 (54.3)	0.016	0.899
Left upper lobe	7 (19.4)	11 (31.4)	1.347	0.246
Left lower lobe	16 (44.4)	13 (37.1)	0.392	0.531
Right lung with infarction	25 (69.4)	26 (74.3)	0.206	0.650
Right upper lobe	9 (25.0)	6 (17.1)	0.657	0.417
Right middle lobe	5 (13.9)	7 (20.0)	0.472	0.492
Right lower lobe	17 (47.2)	23 (65.7)	2.467	0.116
Bilateral lung with infarction	7 (19.4)	7 (20.0)	0.003	0.953
**Number of infarct lesions (Segmental Bronchi)**				
1	22 (61.1)	24 (68.6)	0.433	0.511
≥2	14 (38.9)	11 (31.4)		
**Radiographic features of infarct lesions**				
Hampton's hump	27 (75.0)	27 (77.1)	0.045	0.832
Reversed halo sign	17 (47.2)	10 (28.6)	2.619	0.106
Atelectasis	5 (13.9)	8 (22.9)	0.954	0.329
Semicircular shape	3 (8.3)	2 (5.7)	0.186	0.666
Cavity	1 (2.8)	1 (2.9)	0.000	0.984
Pleural effusion ipsilateral to the infarction	22 (61.1)	14 (40.0)	3.164	0.075

Data are presented as *n* (%).

Comparative analysis revealed no significant differences in either PE risk factors and Wells scores across age groups. Analysis of PE severity classification revealed significant differences between the groups. The older group had a significantly higher proportion of intermediate-high risk cases (33.3 vs. 5.7%, *P* = 0.003), while non-older patients showed a greater prevalence of low-risk classification (19.4 vs. 42.9%, *P* = 0.033). Concordant with this, the simplified Pulmonary Embolism Severity Index (sPESI) revealed a markedly higher proportion of patients with a score of ≥1, indicating a non-low risk status, in the older age group (61.1 vs. 34.3%, *P* = 0.024) (shown in Table 5).

**Table 5:** Comparison of risk factors, Wells score, PE severity classification and sPESI score.

**Variables**	**Age ≥65 years (*n* = 36)**	**Age <65 years (*n* = 35)**	***χ^2^***	***P***
**Risk factor of PE**				
Smoking	13 (36.1)	14 (40.0)	0.114	0.736
Trauma within 4 week	7 (19.4)	8 (22.9)	0.124	0.725
Varicose veins of lower limb	5 (13.9)	5 (14.3)	0.002	0.962
Bed rest > 3 day	6 (16.7)	4 (11.4)	0.402	0.526
Previous lower extremity DVT	2 (5.6)	6 (17.1)	2.383	0.123
Surgical procedure within 4 week	5 (13.9)	3 (8.6)	0.502	0.479
Antiphospholipid syndrome	0 (0)	0 (0)		
**Wells score**				
Low (<2)	14 (38.9)	14 (40.0)	0.009	0.924
Moderate (2–6)	20 (55.6)	16 (45.7)	0.688	0.407
High (>6)	2 (5.6)	5 (14.3)	1.522	0.217
**Risk stratification**				
Low risk	7 (19.4)	15 (42.9)	4.549	0.033
Intermediate low risk	16 (44.4)	15 (42.9)	0.018	0.893
Intermediate high risk	12 (33.3)	2 (5.7)	8.552	0.003
High risk	1 (2.8)	3 (8.6)	1.120	0.290
sPESI score			5.117	0.024
0	14 (38.9)	23 (65.7)		
≥1	22 (61.1)	12 (34.3)		

Data are presented as *n* (%); DVT, deep vein thrombosis; sPESI, simplified Pulmonary Embolism Severity Index.

Comparison of the patient consultation process, diagnosis and treatment of the two groups showed that older patients had higher non-emergency/respiratory first-visit rates than non-older patients (22.2 vs. 2.9%, *P* = 0.014), but lower thrombolytic therapy rates (0.0 vs. 11.4%, *P* = 0.037). No statistically significant differences were observed between the two groups in the number of outpatient/emergency visits, patient-related delays, diagnostic delays, antibiotic usage, hospital length of stay, or mortality (shown in Table 6). Among the eight older patients who initially visited other departments, seven (87.5%) had delayed diagnosis—a rate significantly higher than that of patients who first presented to the emergency/respiratory departments (37.9%; *P* = 0.016).

**Table 6:** Comparison of diagnosis and treatment.

**Variables**	**Age≥65 years (*n* = 36)**	**Age <65 years (*n* = 35)**	***U/χ^2^***	***P***
**Number of outpatient/ED visits**				
1	28 (77.8)	22 (62.9)	1.897	0.168
≥2	8 (22.2)	13 (37.1)		
**Department of first visit**				
Emergency department	21 (58.3)	26 (74.3)	2.018	0.155
Department of respiratory medicine	7 (19.4)	8 (22.9)	0.124	0.725
Other department	8 (22.2)	1 (2.9)	6.012	0.014
Patient-related delay	22 (61.1)	22 (62.9)	0.023	0.880
Delayed diagnosis of PTE	18 (50.0)	16 (45.7)	0.131	0.718
Antibiotic administration	24 (66.7)	25 (71.4)	0.188	0.664
Thrombolysis	0 (0.0)	4 (11.4)	4.360	0.037
Hospital length of stay (day)	11.5 (9.0, 15.0)	10.0 (8.0, 16.0)	567.000	0.467
In-hospital mortality	0 (0.0)	0 (0.0)		

Continuous data are presented as median [IQR]; categorical data are presented as *n* (%). ED, emergency department. The other departments included Vascular Surgery, General Surgery, Neurology, Infectious Diseases, Cardiology, and Nephrology. Patient-related delay, time from symptom onset to first medical contact exceeded 24 h. Delayed diagnosis was defined as ≥24 h elapsed between initial visit and CTPA-confirmed PE diagnosis.

## 4 Discussion

PE is the third most common vascular disease after acute coronary syndrome and stroke, and is one of the major causes of death (1). It is more difficult to diagnose in older patients, as the clinical symptoms and signs of typical PE can also be seen in cardiopulmonary diseases (11, 12). PI is a serious complication of PE, and its imaging manifestations are difficult to distinguish from infectious pneumonia or tumor lesions, which makes the diagnosis more challenging (8, 9). The older population has more underlying diseases and unique pathophysiological characteristics. The clinical symptoms and imaging features of PI may differ from those of young patients. We stratified PI patients by age and analyzed the clinical characteristics of older patients with PE combined with PI, aiming to provide a reference for clinicians to identify and diagnose older PI in the early stage.

Our study showed that 17.1% (71/414) of PE patients developed pulmonary infarction, in line with prior reports (4–7). Consistent with Marjan Islam's findings that young patients face a higher risk of PI post-acute PE, our study revealed a higher PI incidence in non-older patients (24.8%) compared to the older (13.2%) (5). The observed difference may be attributed to the fact that older patients exhibit a significantly higher prevalence of pre-existing cardiovascular and chronic pulmonary diseases, coupled with more developed bronchial collateral circulation compared to younger individuals, and these physiological characteristics may confer a protective effect against PI during PE (13–16). The clinical symptoms of PE combined with PI are mainly dyspnea and chest pain (17). Massimo Miniati proposed the classic “triad” of pulmonary embolism (PE), which includes hemoptysis, pleuritic chest pain, and pleural friction (18). In our study of 71 PI cases, the predominant clinical manifestations were dyspnea (77.5%), pleuritic chest pain (46.5%), and lower limb swelling/pain (40.8%), findings that closely with Miniati's observations (18). Comparison of clinical characteristics between the two groups revealed that older individuals exhibited a significantly lower incidence of pleuritic chest pain and hemoptysis. We hypothesize that the reduced prevalence of chest pain may be attributed to age-related declines in pain sensitivity. However, the underlying mechanisms contributing to the decreased incidence of hemoptysis remain unclear, warranting further pathophysiological investigations for validation.

Our study found that older patients presented with significantly fewer typical ECG changes of right ventricular strain, yet exhibited a higher incidence of pulmonary hypertension compared to the non-older patients. The discrepancy between the frequent presence of pulmonary hypertension in older patients and the relative scarcity of classic ECG patterns (e.g., S1Q3T3) may be attributed to pre-existing or age-related subclinical pulmonary hypertension in older individuals, along with consequent right ventricular (RV) remodeling. This attenuates the electrocardiographic response to acute afterload increase. This contrasts with the more pronounced ECG changes typically observed in younger individuals with previously normal RV function. Secondly, the greater burden of comorbidities such as atrial fibrillation may both confound ECG interpretation, thereby obscuring more specific ischaemic ST-T alterations. Consequently, the hypothesis that age and comorbidity-related factors modify the ECG presentation of acute RV strain warrants further investigation in prospective studies employing broader quantitative ECG parameters. Concurrently, our study observed a higher incidence of elevated BNP and hs-cTnI levels in older patients. Although no statistically significant differences were identified, we consider this marked trend clinically significant. We hypothesize that this phenomenon may stem from the higher prevalence of underlying cardiovascular disease in older patients, leading to more severe cardiac stress and myocardial injury during pulmonary embolism. However, this observation requires validation in future studies with larger sample sizes.

The distribution of infarct lesions in both groups showed that the right lung was more than the left lung, and the incidence rate was higher in the lower lobe, which has also been observed in other reports (5, 19). The predilection for lower lobe infarctions can be explained by pulmonary hemodynamic physiology. According to West's zone theory of pulmonary blood flow (20), gravitational forces and hydrostatic pressure gradients result in approximately 60–70% of normal pulmonary blood flow being distributed to the lung bases in the upright position. Consequently, emboli are more likely to become lodged in these dependent regions. The right lung infarction is more common than the left lung, which is related to the anatomical structure of pulmonary blood flow. The right pulmonary artery is thicker, runs more vertically, and has a smaller angle with the main pulmonary artery. Emboli from the deep veins of the lower limbs are more likely to enter the right pulmonary artery along the direction of blood flow (21, 22). We also observed that PI on CT predominantly manifested as Hampton's hump (76.1%), pleural effusion ipsilateral to the infarction (50.7%), and reversed halo sign (38.0%), findings consistent with previous descriptions by Revel et al. (23, 24). There was no significant difference in the distribution of infarct lesions or imaging features between the two groups, which may indicate that the pathophysiological mechanism of PI is predominantly ischemic necrosis secondary to pulmonary artery obstruction, a process that does not differ substantially across age.

As no validated scoring systems are currently available to assess the likelihood or severity of pulmonary infarction, we applied established pulmonary embolism (PE) scoring criteria in this study. In the study, we found that the risk factors for venous thromboembolism and the pre-test probability as assessed by the Wells' score were comparable between two groups, their initial risk stratification for early mortality diverged significantly. Older patients with PI presented with a markedly more severe risk profile at diagnosis. This is evidenced by their significantly higher prevalence in the intermediate-high risk category and a correspondingly lower proportion in the low-risk stratum of the PE severity classification. The prognostic assessment using the sPESI further corroborated this disparity, with a significantly greater proportion of older patients scoring ≥1 (61.1 vs. 34.3%, *P* = 0.024). Both independent risk assessment frameworks strongly indicate that PE exerts more profound pathophysiological effects in the older, highlighting age itself as a significant determinant of PE prognosis. Collectively, these findings validate the clinical relevance of this cohort study and underscore the necessity for age-specific risk assessment and management strategies in patients with pulmonary embolism.

Analysis of patient pathways indicates that older patients are more likely to initially present to non-emergency/respiratory departments. Subsequent assessments reveal a significantly elevated rate of delayed diagnosis within this cohort, suggesting that non-specialist clinicians encounter a population with more challenging-to-identify PI and exhibit greater deficiencies in recognizing the condition. Regarding treatment, intergroup comparative analysis demonstrates that although older PI patients exhibit a higher proportion of moderate-to-high risk cases, the rate of thrombolytic therapy remains lower than that observed in younger patients. Systemic thrombolysis was administered to four patients, all of whom were in the younger group. This discrepancy may be attributed to a higher prevalence of contraindications to thrombolysis among the older or to greater clinician reluctance stemming from concerns about bleeding risks in this population. This treatment pattern likely reflects enhanced clinical caution in managing older patients, in whom conservative management is often favored due to a less favorable risk-benefit profile. These observations underscore that therapeutic decision-making for older patients with PI is complex and necessitates an individualized approach that carefully weighs the risk of bleeding against the severity of the condition. Antibiotic utilization rates were comparable between the two groups, with no statistically significant difference (66.7 vs. 71.4%, *P* = 0.664). This indicates that pulmonary infarction is frequently misdiagnosed as pneumonia in both older and non-older patients, with elevated antibiotic usage being a general phenomenon across our entire study cohort rather than a bias specific to the older group. This finding underscores the diagnostic challenge of clinically distinguishing pulmonary infarction from pneumonia, particularly when patients present with non-specific respiratory symptoms and radiological opacities. The high rate of empirical antibiotic treatment reflects a common clinical dilemma in acute care settings. Consequently, enhancing recognition of radiological features suggestive of pulmonary infarction is crucial for improving diagnostic accuracy and guiding appropriate treatment decisions.

Our study has several limitations that should be considered when interpreting the results. First and foremost, the retrospective, single-center design inherently carries a risk of selection bias and limits the generalizability of our findings. The relatively limited sample size further constrains the statistical power of the analysis; as such, our findings should be regarded as preliminary and hypothesis-generating, requiring validation in larger, multicenter cohorts. Second, several important analyses could not be performed. In our institutional practice, CTPA is often directly performed for patients with a high clinical suspicion of PE, bypassing routine chest X-rays. Consequently, the available chest X-ray data are sparse and unlikely to yield reliable conclusions, precluding a meaningful analysis of its role in the early diagnosis or differential diagnosis of PE. Furthermore, we did not perform a quantitative analysis correlating embolus size with the extent of parenchymal opacity, which represents a pertinent and valuable direction for investigation. Therefore, we will undertake a dedicated effort in future studies to systematically collect and analyze these specific data points. Third, a significant shortcoming is the lack of long-term follow-up data. Our analysis was limited to the index hospitalization, and we were unable to assess critical long-term outcomes such as mortality, recurrence, functional recovery, or the incidence of chronic thromboembolic pulmonary hypertension (CTEPH). Therefore, while we identified initial differences in clinical presentation between age groups, whether these differences translate into divergent long-term prognoses remains an critical unanswered question. Understanding if PI itself confers a different long-term outcome compared to embolism alone warrants dedicated prospective studies with extended follow-up. In subsequent research, we will expand the sample size and incorporate more comprehensive data to enhance the statistical reliability. We plan to perform multicenter prospective studies to create an early prediction model for pulmonary infarction in older patients, optimizing clinical management and supporting clinical decision-making.

In summary, older patients with PI have fewer typical symptoms like pleuritic chest pain, hemoptysis, or ECG changes, but a high proportion of comorbid cardiovascular/ecrebrovascular diseases, and a higher rates of mismatch in the first visiting department. These findings suggest that diagnosing PI in older patients is more challenging. It is essential for clinicians to provide more attention to this particular group in order to avoid missed or misdiagnosed cases. The classic clinical presentation and overall clinical significance of PI are not fully characterized. Based on the findings of this study, we recommend maintaining a low threshold for CTPA in patients presenting with peripheral ground-glass opacities, Hampton's hump, or ipsilateral pleural effusion on imaging, particularly when accompanied by symptoms such as dyspnoea, pleuritic chest pain, lower limb oedema or pain, or haemoptysis. This recommendation is especially pertinent for older patients with a Wells score exceeding 6, or those with concomitant pulmonary hypertension or right ventricular dysfunction. Furthermore, clinicians should maintain heightened vigilance for pulmonary infarction in older patients presenting with unexplained syncope or deteriorating cardiac function, even in the absence of typical symptoms such as pleuritic chest pain or haemoptysis. These recommendations are intended to provide clear guidance for clinical practice and to improve the early diagnosis of pulmonary infarction in high-risk older populations.

## Data Availability

The raw data supporting the conclusions of this article will be made available by the authors, without undue reservation.
